# A theory-based online health behaviour intervention for new university students (U@Uni): results from a randomised controlled trial

**DOI:** 10.1186/1471-2458-14-563

**Published:** 2014-06-05

**Authors:** Tracy Epton, Paul Norman, Aba-Sah Dadzie, Peter R Harris, Thomas L Webb, Paschal Sheeran, Steven A Julious, Fabio Ciravegna, Alan Brennan, Petra S Meier, Declan Naughton, Andrea Petroczi, Jen Kruger, Iltaf Shah

**Affiliations:** 1 Department of Psychology, University of Sheffield, Western Bank, Sheffield S10 2TP, UK; 2 School of Psychological Sciences, University of Manchester, Oxford Road, Manchester M13 9PL, UK; 3 Department of Computer Science, University of Sheffield, Regent Court, Sheffield S1 4DA, UK; 4 School of Psychology, University of Sussex, Falmer BN1 9QH, UK; 5 Psychology Department, University of North Carolina, 323 Davie Hall, Chapel Hill NC 27599-3270, USA; 6 School of Health and Related Research, University of Sheffield, Regent Court, Sheffield S1 4DA, UK; 7 School of Life Science, Kingston University, Penrhyn Road, Kingston upon Thames KT1 2EE, UK

**Keywords:** Young adults, Internet, Self-affirmation, Theory of planned behaviour, Implementation intentions, Alcohol, Fruit and vegetables, Exercise, Smoking

## Abstract

**Background:**

Too few young people engage in behaviours that reduce the risk of morbidity and premature mortality, such as eating healthily, being physically active, drinking sensibly and not smoking. This study sought to assess the efficacy and cost-effectiveness of a theory-based online health behaviour intervention (based on self-affirmation theory, the Theory of Planned Behaviour and implementation intentions) targeting these behaviours in new university students, in comparison to a measurement-only control.

**Methods:**

Two-weeks before starting university all incoming undergraduates at the University of Sheffield were invited to take part in a study of new students’ health behaviour. A randomised controlled design, with a baseline questionnaire, and two follow-ups (1 and 6 months after starting university), was used to evaluate the intervention. Primary outcomes were measures of the four health behaviours targeted by the intervention at 6-month follow-up, i.e., portions of fruit and vegetables, metabolic equivalent of tasks (physical activity), units of alcohol, and smoking status.

**Results:**

The study recruited 1,445 students (intervention *n* = 736, control *n* = 709, 58% female, *Mean* age = 18.9 years), of whom 1,107 completed at least one follow-up (23% attrition). The intervention had a statistically significant effect on one primary outcome, smoking status at 6-month follow-up, with fewer smokers in the intervention arm (8.7%) than in the control arm (13.0%; Odds ratio = 1.92, *p* = .010). There were no significant intervention effects on the other primary outcomes (physical activity, alcohol or fruit and vegetable consumption) at 6-month follow-up.

**Conclusions:**

The results of the RCT indicate that the online health behaviour intervention reduced smoking rates, but it had little effect on fruit and vegetable intake, physical activity or alcohol consumption, during the first six months at university. However, engagement with the intervention was low. Further research is needed before strong conclusions can be made regarding the likely effectiveness of the intervention to promote health lifestyle habits in new university students.

**Trial registration:**

Current Controlled Trials, ISRCTN67684181.

## Background

Young people are at risk of developing serious health problems and diseases, such as cancer, heart and circulatory disease, obesity and type 2 diabetes, in the future due to their current lifestyle choices [1]. A recent health survey found that few young people in the UK perform recommended health behaviours that reduce these health risks, with only 20% of 16-24 year olds reporting that they eat five portions of fruit and vegetables per day, less than 50% reporting that they meet weekly physical activity guidelines, 40% reporting that they exceed daily recommended alcohol limits, and 25% reporting that they smoke tobacco [2]. Given the high percentages in some of these health risks, it is likely that many young people simultaneously engage in a variety of health-compromising behaviours. There is therefore a need for multi-behaviour health interventions aimed at young people. There is some evidence that multi-behaviour health interventions can have positive effects on lifestyle habits [3]. For example, successful multi-behaviour health interventions to promote both exercise and healthy diets have been reported for school children [4] and undergraduates [5]. This paper reports the results of a randomised controlled trial (RCT) to test the efficacy of a theory-based online intervention to promote healthy lifestyle habits (U@Uni) delivered during the transition from school to university [6]. To the best our knowledge, this is first test of a multi-behaviour health intervention delivered during this transition.

The transition from school to university may represent an ideal opportunity to deliver such interventions. First, it is possible to target a large proportion of young people in the UK. More than 350,000 students aged 20 or under start university each year, representing approximately 40% of school leavers [7]. Second, major life transitions, such as the move to university, represent a critical or “teachable” moment to intervene in order to promote healthy lifestyle habits. The inherent change in young peoples’ environmental context, including the disruption of established peer networks, means that health beliefs and behaviours are likely to be in a state of flux and therefore more amenable to change [8,9]. Moreover, moving to a new location has been found to promote changes in behaviours such as quitting smoking [10].

Evidence indicates that the use of theory in the design of health behaviour interventions increases their efficacy [3]. The current intervention therefore included three theory-based techniques to promote health behaviour change. First, a self-affirmation manipulation was included to reduce defensive processing of health messages [11]. Second, theory-based messages were designed to increase motivation to adopt healthy behaviours [12]. Third, participants were prompted to form implementation intentions to help them to translate their intentions to change into behaviour [13].

The intervention was delivered online via a website and mobile app. The use of digital technologies to deliver a health behaviour intervention has a number of advantages [3]. In particular, the use of digital technologies means that it is possible to (a) deliver interventions to large numbers of people at relatively low cost, (b) ensure that the intervention is accessible 24 hours a day, so is available at critical moments, and (c) increase engagement through the use of interactive methods such as video streaming and sharing resources. In addition, the use of digital technologies may be particularly relevant to young people, who are the prime users of such technology [14,15]. A recent meta-analysis confirmed the potential of online health behaviour interventions, reporting a small but significant effect size (*d* = 0.16) on health behaviour [3].

The efficacy of the U@Uni health behaviour intervention, delivered shortly before students started university, was assessed using a randomised controlled design with follow-up 1 and 6-months after starting university. The two arms of the RCT were (i) an online intervention targeting four health behaviours and (ii) a measurement only control, with approximately 50% of participants randomly allocated to each condition. Full details of the intervention are provided in an earlier paper reporting the study protocol [6].

## Method

### Participants and procedure

**Figure 1:**
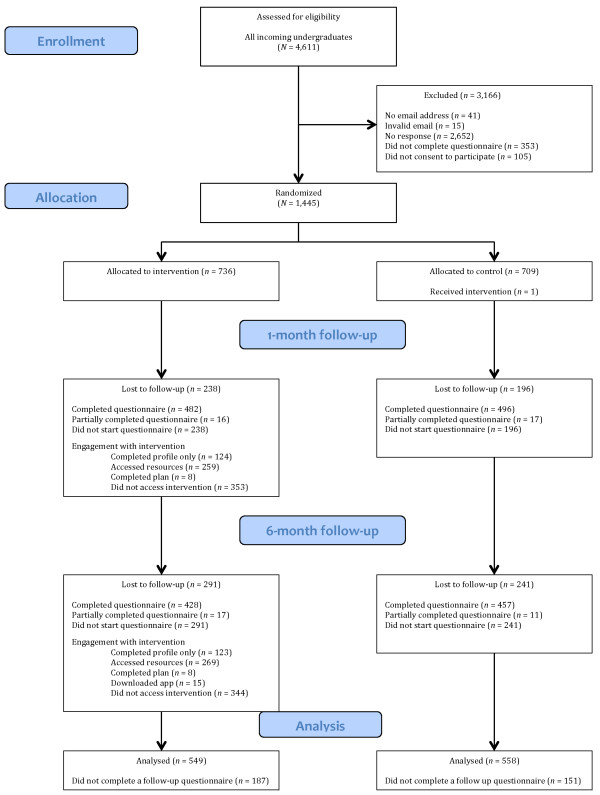
Flow of participants through the trial.

Two weeks before starting university (September 2012), incoming undergraduate students to the University of Sheffield (all of whom were eligible to participate) (*N* = 4,611) were sent an email inviting them to take part in the U@Uni study, with a link to an online questionnaire with baseline measures of demographics, beliefs, and behaviour. *N* = 1,445 incoming students (*Mean* age = 18.9 years; 58% female) completed the baseline questionnaire and were randomly allocated to the intervention (*n* = 736) and control arms (*n* = 709) using the random function on SurveyGizmo [16]. As detailed in the study protocol [6], we assumed a 50% response rate to the initial email invite and 40% attrition at 6-month follow-up. With an anticipated 4,000 eligible participants, this would result in a final sample of 1,200 for the proposed analyses. It was calculated that the trial would have at least 80% power to detect a small effect size (*d* = 0.20) at a two-tailed significance level of .0127 (adjusted for multiple primary outcomes). Informed consent was obtained on the first page of the questionnaire (participants indicated their consent to participate by clicking a button before they were permitted to proceed to the questionnaire). See Figure 1 for the flow of participants through the trial and Table 1 for details of the baseline sample.

**Table 1:** Baseline characteristics of the sample

**Variable**	**Control**			**Intervention**		
	**% or mean**	***SD***	***N***	**% or mean**	***SD***	***N***
**Demographic**						
Nationality						
UK	75.74	-	537	74.46	-	548
Non UK	24.26	-	172	25.54	-	188
Ethnicity						
White British	67.42	-	476	65.98	-	483
White other	5.95	-	42	6.97	-	51
Mixed	3.97	-	28	2.46	-	18
Asian and Asian British	8.64	-	61	8.61	-	63
Black and Black British	2.27	-	16	2.46	-	18
Chinese	10.48	-	74	12.16	-	89
Other	1.27	-	9	1.37	-	10
Gender						
Female	55.15	-	391	61.55	-	453
Male	44.85	-	318	38.45	-	283
Age	19.04	2.91	709	18.76	1.99	736
**Fruit and vegetable intake**						
Mean portions	6.36	4.96	669	6.67	5.17	701
**Physical activity**						
METS	3402.37	5101.72	688	3140.11	3861.42	709
Mean hours sitting	344.36	179.09	609	336.17	171.42	641
**Alcohol consumption**						
Mean total units in last 7 days	11.88	18.54	708	11.17	18.72	736
Mean number of days binge drinking in last 7 days (drinkers only)	1.00	1.04	447	1.04	1.14	425
Mean alcohol objective (FAEE)	2.07	2.02	54	2.08	2.56	54
**Smoking**						
Has smoked	37.24	-	264	37.23	-	274
Has never smoked	62.76	-	445	62.77	-	462
Current smokers	11.99	-	85	11.28	-	83
Not a current smoker	88.01	-	624	88.72	-	653
Cigarettes smoked per week	3.19	14.12	496	1.88	10.95	483
Smoking objective (cotinine)	.48	.49	54	.45	.42	54
Smoking objective (nicotine)	7.19	18.67	54	9.49	19.16	54
**Other outcomes**						
***EQ-5D-3L***						
Mean health index scores from EQ-5D-3L (VAS)	.90	.14	708	.91	.14	735
Mean health index score from EQ-5D-3L (TTO)	.92	.13	708	.92	.14	735
Mean EQ-5D-3L visual analogue scale	78.21	13.61	706	78.11	15.85	730
***Recreational drugs***						
Have taken recreational drugs (SSC measure)^1^	18.73	Â± 9.54	132	16.49	Â± 9.56	121
Have not taken recreational drugs (SSC measure)	81.27		575	83.51		613
Have taken recreational drugs (biochemical measure)	39.53	-	17	45.71	-	16
Have not taken recreational drugs (biochemical measure)	60.47	-	26	54.29	-	19
***BMI***						
Mean BMI self-report	22.06	3.71	662	22.24	3.81	695
Mean BMI objective	22.28	3.85	54	22.06	3.88	54
**Social cognition variables**						
Fruit & veg						
Self-efficacy	5.71	1.48	703	5.80	1.36	733
Perceived control	5.51	1.47	703	5.53	1.45	733
Intention	5.24	1.47	703	5.39	1.45	733
Physical activity						
Self-efficacy	5.97	1.37	707	6.03	1.31	734
Perceived control	5.66	1.46	707	5.58	1.46	734
Intention	5.87	1.36	707	5.86	1.37	734
Binge drinking						
Self-efficacy	5.48	2.15	705	5.38	2.20	732
Perceived control	5.94	1.55	705	6.05	1.43	732
Intention	3.20	1.96	705	3.12	1.99	732
Smoking						
Self-efficacy	4.94	2.48	706	4.90	2.51	733
Perceived control	6.39	1.45	706	6.51	1.27	733
Intention	1.56	1.41	706	1.52	1.30	733

^1^Includes confidence interval. Notes. METS = metabolic equivalent of task, FAEE = fatty acid ethyl esters, VAS = visual analogue scale technique, TTO = time trade-off technique, SSC = single count method, BMI = body mass index.

Participants assigned to the intervention arm were directed to the U@Uni website and asked to complete a profile page that contained the self-affirmation manipulation. After completing their profile, participants were asked to sign in to the website and view the online resources, which included theory-based messages (i.e., text, videos and links to further information) relevant to each of the four targeted health behaviours and a planner that contained instructions to form implementation intentions. Participants were able to selectively access information that was of interest to them and also had the opportunity to access more detailed information (using links to further information or via a search function).

Intervention participants were emailed prior to the start of the second university semester and invited to download a smartphone app designed for the Android operating system from the U@Uni website. The app and the website were accessible to intervention participants throughout the academic year.

All participants were asked to complete a follow-up questionnaire 1-month (October 2012) and 6-months (March 2013) after starting university. Participants were paid £10 for completing all three questionnaires and were entered into a £100 prize draw for each questionnaire they completed.

Participants were also sent emails when they started university and 6-months later inviting them to participate in an additional study on the biochemical markers of health behaviour. A sample of 108 students (Intervention *n* = 54, Control *n* = 54, *Mean* age = 19.58 years, *SD* = 3.83) was recruited to this additional study at baseline and 78 of these also provided a hair sample at 6-month follow-up (Intervention *n* = 35, Control *n* = 43).

Ethical approval for the study was obtained from the Department of Psychology Research Ethics Committee at the University of Sheffield.

### Intervention materials

Full details of the intervention are provided in the protocol paper [6].

#### Self-affirmation task

The self-affirmation manipulation was adapted from an existing value-affirmation task [17] and embedded in a profile page. Participants were presented with a list of eight commonly held personal values (sense of humour, academic achievement, relations with family and friends, social skills, spontaneity, artistic skills/aesthetic appreciation, religion/faith/spirituality, and respect/decency/manners) and asked to select their most important value (or provide their own) and to briefly explain why the value was important to them.

#### Theory-based messages

Theory-based persuasive messages were developed to encourage regular exercise and fruit and vegetable intake, and to discourage binge drinking and smoking. The messages were based on the Theory of Planned Behaviour [18] and developed on the basis of formative work that identified the key behavioural, normative and control beliefs associated with intentions to perform each of the four health behaviours in new university students [see [19], for details of the health message development process]. The messages included a mixture of text and videos, as well as links to other relevant material.

#### Implementation intentions

The planner comprised a series of drop down menus that helped participants to form implementation intentions by asking them to identify (i) a good opportunity to act on their intentions (e.g., when tempted to binge drink) and (ii) a suitable response to their identified opportunity (e.g., to remind themselves that they have lectures tomorrow) for each of the four targeted health behaviours. The planner also allowed participants to type in their own opportunities and responses. The plans were stored in a ‘plan repository’ and participants were also able to opt to have a reminder of each plan emailed to them.

### Measures

The four primary outcome measures were (i) portions of fruit and vegetables per day, (ii) physical activity in the last week, (iii) alcohol consumption in the last week, and (iv) smoking status at 6-month follow-up. A range of secondary outcome measures was also assessed as detailed below. Unless indicated, all of the measures were taken at baseline as well as at 1 and 6-month follow-up.

#### Fruit and vegetable intake

Fruit and vegetable intake (portions per day) was measured with items based on the Health Survey for England (HSE) [2]. Participants were asked to think about the preceding day and indicate whether they had eaten any of nine different types of fruit and vegetables (e.g., “Did you eat any salad yesterday?”) and if so how much of each type they had eaten (e.g., “How many cereal bowls of salad did you eat yesterday?”).

#### Physical activity

The Short Form of International Physical Activity Questionnaire (IPAQ-SF) was used to assess levels of physical activity [20]. Respondents were asked to indicate how many times, and for how long, they had engaged in vigorous exercise (defined as “activities that take hard physical effort and make you breathe much harder than normal”), moderate exercise (defined as “activities that take moderate physical effort and make you breathe somewhat harder than normal”) and walking in the past 7 days. Responses were converted into METs (metabolic equivalent of task) to provide a total IPAQ score. An additional question asked about sedentary activity. Data on use (membership and number of visits) of the university sports facilities were collected from automatic records kept by the university sports centre (users have to swipe a card when they attend).

#### Alcohol

Alcohol consumption was assessed using items from the General Lifestyle Survey [21] to provide a measure of units of alcohol per week and number of binge sessions per week (i.e., participants were asked to indicate on which days they had drunk alcohol in the last 7 days and the type and amount of alcohol that they drank on each day). The Alcohol Use Disorders Identification Test (AUDIT) [22] was used at the 6-month follow-up to assess hazardous and harmful patterns of alcohol use.

#### Smoking

Items based on the HSE were used to assess participants’ current smoking status and the typical number of cigarettes/amount of tobacco that they smoked [2].

#### Health status

The EQ-5D-3L [23], a short standardized measure of health status, was used to assess levels of severity (no problems/some or moderate problems/extreme problems) in five dimensions: mobility, self-care, usual activities, pain/discomfort, and anxiety/depression. The measure provides a descriptive profile and a single index value for health status and is recommended as the measure of health-related quality of life for health economic evaluations in the UK [24].

#### Recreational drug use

A Single Sample Count Method [25] was used to estimate the prevalence of recreational drug use in the sample. Respondents were asked to indicate the number of “yes” answers (0 or 5, 1, 2, 3, 4) to five questions – four of which have a 50% population prevalence (e.g., odd or even date of birth) and one of which was about their use of recreational drugs. In this way it was possible to estimate the prevalence of recreational drug use in the sample without being able to identify whether individual participants do or do not use recreational drugs, as 50% of the sample would answer “yes” answers to each of the four questions. This Single Sample Count Method has been shown to encourage accurate reporting of behaviours that are illegal and could be regarded as socially undesirable [26].

#### BMI

Participants recorded their height and weight, from which their BMI was calculated.

#### Health services usage

Participants were asked to report their use of health services (e.g., GP visits, hospitalizations) at the 6-month follow-up.

#### Academic performance

Average exam marks and registration status (i.e., registered level one passed, registered level one failed, withdrawn, leave of absence) were used to assess academic performance at the end of the academic year.

#### Social cognitive variables

Measures of social cognitive variables for each behaviour were included. Each variable was measured with one item per behaviour, except attitude, which was measured with two items. Measures of intention (e.g., “I intend to engage in regular exercise at university”), self-efficacy (e.g., “If I wanted, I could easily engage in regular exercise at university”), and perceived control (e.g., “Whether or not I engage in regular exercise at university is under my control”) were taken at all time points. Measures of attitude (e.g., “Engaging in regular exercise at university would be… good/bad”), subjective norms (e.g., “Most people who are important to me think I should/should not engage in regular exercise at university) and planning (e.g., “To what extent do you have a detailed plan about how to engage in regular exercise at university?”) were taken at the 1 and 6-month follow-ups.

#### Engagement with the digital intervention

Measures of engagement with the intervention, for both the website and the mobile app, were (i) completion of the self-affirmation task (i.e., profile page), (ii) whether or not participants accessed the theory-based messages and (iii) the number of implementation intentions that were formed.

#### Biochemical measures

Participants recruited to the additional study on the biochemical markers of health behaviour provided a hair sample (3 cm long) that was liquefied and analysed for biochemical markers of various health behaviours related to alcohol consumption, cigarette smoking, and recreational drug use. Following extraction procedures, markers of alcohol (fatty acid ethyl esters [FAEE]) and cigarettes (nicotine, cotinine) were quantified using liquid chromatography with tandem mass spectrometric detection (LC-MS/MS). In addition, evidence for recreational drug use was detected by screening for commonly used drugs and their metabolites. These included: amphetamine, 3,4-methylenedioxyamphetamine (MDA), 3,4-methylenedioxy-N-methylamphetamine (MDMA), ephedrine, mephedrone, tetrahydrocannabinol (THC), cocaine, heroin, lysergic acid diethylamide (LSD), phencyclidine (PCP) and ketamine. Morphine, codeine, hydromorphone and hydrocodone were treated separately owing to their potential medical use (i.e., as a pain reliever or cough suppressant). A 6430 triple quadruple mass spectrometer (made by Agilent Technologies UK) was employed with a dynamic-multiple reaction monitoring-liquid chromatography mass spectrometry (DYN-MRM-LC-MS/MS) method. Participants recruited to this additional study also had their height and weight measured to calculate an objective measure of BMI.

### Statistical analysis

Analysis of the 6-month data was conducted using an intention-to-treat approach (i.e., data were included from all participants who completed at least one follow-up survey); missing data at 6-months were imputed from the 1-month follow-up data by carrying the last observation forward [27,28]^a^. Analysis of the 1-month data was based only on participants who completed the 1-month survey.

A series of analyses of covariance (ANCOVA) and logistic regression analyses were used to assess the impact of the intervention on performance of the targeted behaviours, controlling for corresponding baseline scores, gender, age and nationality (i.e., UK or non-UK). For primary outcomes, the bonferroni correction was used; thus statistical significance was declared if any of the primary endpoints were significant at .0127 to account for multiple tests.

The impact of the intervention on secondary outcomes (i.e., health behaviours at 1-month follow-up, social cognitive variables, health status, recreational drug use, BMI, health services usage, academic performance, use of university sports facilities and biochemical measures) was assessed using a similar analysis strategy, i.e., using ANCOVAs and logistic regression analyses that controlled for corresponding baseline scores (where available), gender, age and nationality. As these were secondary outcomes no adjustments were made for multiple tests. The analyses were repeated to (i) assess the effect of engagement with the intervention (per protocol analyses) and (ii) to assess the effect of moderators (with dichotomised moderators as additional IVs). Additional analyses were conducted to compare dropouts and completers on the baseline measures.

## Results

### Randomisation check

There were no differences between participants in the intervention and control arms in baseline measures of the four health behaviours. Gender and age did, however, differ between the two arms (see Table 1), with more females and younger participants in the intervention arm than in the control arm.

### Primary outcomes

At 6-month follow-up, the intervention and control arms had statistically significant differences in the number of current smokers (B = .65, *SE* = .25, *p* = .010); 8.70% of participants in the intervention arm reported that they were current smokers compared to 13.01% of participants in the control arm. More detailed analyses revealed that, of the 480 non-smokers in the intervention arm at baseline, 14 (2.92%) were smokers at 6-month follow-up. In contrast, of the 489 non-smokers in the control arm at baseline, 27 (5.52%) were smokers at 6-month follow-up. In addition, of the 60 smokers in the intervention arm at baseline, 27 (45.00%) were non-smokers at 6-month follow-up. In contrast, of the 64 smokers in the control arm at baseline, 19 (29.69%) were non-smokers at 6-month follow-up.

Fruit and vegetable intake, physical activity, and alcohol consumption at 6-month follow-up did not differ significantly between the two arms (see Table 2).

**Table 2:** Estimated marginal means, percentages, sample sizes, standard deviations, differences, p values and effect sizes for primary and secondary outcomes

	**1-month follow-up**									**6-month follow-up**								
**Variable**	**Control**			**Intervention**						**Control**			**Intervention**					
	**% or Mean**	***SD***	***n***	**% or Mean**	***SD***	***n***	**Diff**	***p***	***d***	**% or Mean**	***SD***	***n***	**% or Mean**	***SD***	***n***	**Diff**	***p***	***d***
**Fruit and vegetable intake**																		
Mean portions	5.47	4.26	453	6.02	4.18	436	.55	.053	0.13	5.72	4.98	512	5.61	4.89	495	-.11	.708	-0.02
**Physical activity**																		
Mean METS	2563.39	2162.88	471	2755.63	2169.40	452	192.24	.179	0.09	3316.10	5143.79	526	3350.52	5144.16	513	34.42	.914	0.01
Mean hours sitting	400.55	150.49	388	396.19	150.44	372	-4.36	.890	0.03	408.12	158.25	450	412.24	158.30	435	4.12	.699	-0.03
Member of Sport Sheffield										56.52	-	325	54.92	-	318	-1.60	.392	-0.04
Not a member of Sport Sheffield										43.48	-	250	45.08	-	261			OR 1.11
Mean Sport Sheffield attendance										8.15	15.50	325	9.92	15.34	318	1.77	.149	0.15
**Alcohol consumption**																		
Mean units in last 7 days	13.86	16.89	507	12.55	17.06	491	.02	.222	0.08	13.41	19.65	547	13.01	19.75	540	-.40	.737	0.02
Mean number of days binge drinking in last 7 days (drinkers only)	1.33	.99	271	1.31	.92	234	-.02	.901	0.02	1.16	.89	319	1.16	.85	288	.00	.973	0.00
AUDIT																		
Mean consumption										6.45	2.60	400	6.32	2.52	376	-.13	.480	0.05
Mean dependency										.93	1.39	393	.88	1.34	366	-.05	.597	0.04
Mean problems										2.26	2.60	393	2.05	2.49	366	-.21	.256	0.08
Mean alcohol objective (FAEE)										2.55	4.59	43	2.80	4.61	34	.25	.816	-0.05
**Smoking**																		
Has smoked	42.37	-	211	40.41	-	196	-1.96	.849	0.04	47.74	-	264	46.30	-	250	-1.44	.776	0.03
Has never smoked	57.63	-	287	59.59	-	289	1.96		OR 1.04	52.26	-	289	53.70	-	290	1.44		OR 1.05
Smoked since attending University	25.96	-	129	24.33	-	118	-1.63	.578	0.05	35.51	-	196	35.93	-	194	.42	.878	-0.01
Not smoked since starting University	74.04	-	368	75.67	-	367	1.63		OR 1.09	64.49	-	356	64.07	-	346	-.42		OR 0.98
Current smoker	11.45	-	57	9.07	-	44	-2.38	.333	0.14	13.02	-	72	8.70	-	47	-4.32	.010	0.25
Not a current smoker	88.55	-	441	90.93	-	441	2.38		OR 1.36	86.98	-	481	91.30	-	493	4.32		OR 1.92
Mean cigarettes smoked per week	2.74	5.57	496	2.35	5.71	483	-.39	.286	0.07	3.24	6.81	552	2.70	6.73	538	-.54	.181	0.08
Mean smoking objective (cotinine)										.37	.33	43	.51	.35	34	.14	.081	-0.41
Mean smoking objective (nicotine)										7.24	21.44	43	12.85	21.52	34	5.61	.266	-0.26
**Other outcomes**																		
***EQ-5D-3L***																		
Mean health index scores from EQ-5D-3L(VAS)	.91	.22	495	.91	.22	482	0.00	.957	0.00	.90	.12	542	.91	.23	530	.01	.452	0.05
Mean health index scores from EQ-5D-3L (TTO)	.92	.22	495	.92	.22	482	0.00	.728	0.00	.92	.23	542	.92	.23	530	.00	.739	0.00
Mean EQ-5D-3L visual analogue scale	77.63	12.45	494	76.66	12.45	477	-.97	.224	-0.08	77.69	13.01	540	77.17	13.05	524	.01	.508	-0.04
***Recreational drug use***																		
Have taken recreational drugs (SSC measure)^1^	9.52	Â±10.74	47	22.57	Â±12.11	109	13.05	<.001	-0.57	8.27	Â±11.12	38	24.36	Â±12.7	105	16.09	<.001	-0.70
Have not taken recreational drugs (SSC measure)	90.48		450	77.43		373	-13.05		OR 2.04	91.73		418	75.64		324	-16.09		OR 2.39
Have taken recreational drugs (biochemical measure)										41.86	-	18	45.71	-	16	3.85	.491	-0.09
Have not taken recreational drugs (biochemical measure)										58.14		25	54.29	-	19	-3.85		OR 1.29
***BMI***																		
Mean BMI	21.97	2.96	448	22.22	3.15	440	.25	.230	-0.08	22.15	2.23	499	22.12	2.22	494	-.03	.870	0.01
Mean objective BMI										22.01	2.90	100	22.27	2.86	91	.01	.550	-0.09
***Health service usage***^***2***^																		
Mean times visited GP in last 6 months										1.33	1.49	455	1.18	1.66	429	.01	.169	0.10
Alcohol intervention offered by GP										.39	-	1	.82	-	2	.43	.648	-0.41
Alcohol intervention not offered by GP										99.61	-	253	99.18	-	241	-.43		OR 1.68
Attended alcohol intervention										0	-	0	0	-	0	.00	-	
Did not attend alcohol intervention										100	-	1	100	-	2	.00		
Mean times visited A&E										.10	.43	452	.10	.41	426	.00	.882	0.00
Mean times admitted to A&E										.19	.52	42	.25	.51	40	.06	.622	-0.12
Mean times required an ambulance										.03	.21	429	.03	.20	404	.02	.802	0.00
Mean times admitted to hospital										.04	.21	450	.06	.21	425	.02	.253	-0.10
Mean elective admissions to hospital										.34	.69	13	.68	.67	20	.34	.181	-0.50
Mean non-elective admissions to hospital										1.02	.66	12	.44	.63	20	-.58	.020	0.90
Mean other times visited hospital (not incl. above)										.25	.85	447	.22	.82	424	-.03	.642	0.04
Academic achievement																		
Mean grade of academic year										62.49	8.99	481	62.35	9.13	473	-.14	.814	-0.02
Progressed from level one										96.54		446	95.45		435	-.09	.810	-0.01
Did not progress										3.46	-	16	3.55		16	.09		OR 1.01
**Social cognition variables**																		
Fruit and vegetables																		
Mean descriptive norm	2.67	1.10	488	2.63	1.09	478	-.04	.684	-0.04	5.61	1.62	534	5.37	1.84	527	.24	.027	-0.14
Mean injunctive norm	6.05	1.10	488	5.93	1.08	478	-.12	.116	-0.11	3.18	1.62	534	3.43	1.61	527	.25	.017	0.15
Mean perceived control	5.63	1.33	488	5.65	1.31	478	.02	.785	0.02	5.66	1.39	534	5.85	1.38	527	.19	.018	0.14
Mean self-efficacy	5.50	1.55	488	5.49	1.53	478	-.01	.944	-0.01	5.58	1.39	534	5.72	1.38	527	.14	.106	0.10
Mean intention	5.39	1.10	488	5.31	1.09	478	-.08	.270	-0.07	5.52	1.16	534	5.47	1.15	527	-.05	.442	-0.04
Mean plan	4.85	1.55	488	4.78	1.53	478	-.07	.474	-0.05	5.09	1.39	534	5.13	1.38	527	.04	.628	0.03
Mean attitude	6.22	.88	488	6.20	.87	478	-.22	.652	-0.02	6.65	.69	534	6.59	.69	527	-.06	.208	-0.09
Physical activity																		
Mean descriptive norm	3.75	1.33	493	3.78	1.31	480	.03	.637	0.02	5.71	1.39	539	5.65	1.38	527	-.06	.530	-0.04
Mean injunctive norm	6.04	1.11	493	5.99	1.31	480	-.05	.550	-0.04	4.10	1.63	539	4.20	1.61	527	.10	.288	0.06
Mean perceived control	5.80	1.11	493	5.93	1.10	480	.13	.078	0.12	5.83	1.16	539	5.96	1.15	527	.13	.069	0.11
Mean self-efficacy	5.62	1.33	493	5.82	1.31	480	.20	.020	0.15	5.78	1.39	539	5.83	1.38	527	.05	.523	0.04
Mean intention	5.63	1.11	493	5.64	1.10	480	.01	.921	0.01	5.67	1.16	539	5.74	1.15	527	.07	.306	0.06
Mean plan	4.94	1.55	493	5.04	1.53	480	.08	.338	0.06	5.14	1.63	539	5.13	1.61	527	-.01	.944	-0.01
Mean attitude	6.10	.89	493	6.18	.88	480	.08	.119	0.09	6.63	.70	539	6.62	.69	527	.01	.948	-0.01
Binge drinking																		
Mean descriptive norm	5.70	1.11	491	5.59	1.31	479	-.11	.177	0.09	2.79	1.85	537	3.00	1.83	525	.21	.059	-0.06
Mean injunctive norm	2.48	1.33	491	2.47	1.31	479	-.01	.974	0.01	5.10	1.62	537	4.93	1.83	525	-.17	.110	0.10
Mean perceived control	6.24	1.11	491	6.13	1.09	479	-.11	.171	-0.01	6.27	1.16	537	6.14	1.15	525	-.13	.097	-0.10
Mean self-efficacy	5.70	1.55	491	5.66	1.53	479	-.04	.704	-0.03	5.81	1.39	537	5.69	1.38	525	-.12	.194	-0.09
Mean intention	3.18	1.11	491	3.16	1.31	479	-.02	.824	0.02	3.13	1.39	537	3.07	1.37	525	-.06	.457	0.04
Mean plan	4.66	1.99	491	4.45	1.97	479	-.21	.115	-0.11	4.89	2.09	537	4.50	2.06	525	.39	.003	-0.19
Mean attitude	2.50	.89	491	2.52	.88	479	.02	.819	-0.02	1.83	.93	537	1.91	1.15	525	.09	.207	-0.08
Smoking																		
Mean descriptive norm	4.19	1.33	488	4.05	1.31	479	.14	.113	0.11	1.73	1.39	537	1.87	1.37	525	.14	.105	-0.10
Mean injunctive norm	1.34	.88	488	1.35	.88	479	.01	.751	-0.01	3.63	1.62	537	3.57	1.60	525	-.06	.593	0.04
Mean perceived control	6.61	1.10	488	6.58	1.09	479	.03	.666	-0.03	6.55	1.16	537	6.51	1.15	525	-.04	.518	-0.03
Mean self-efficacy	5.36	1.77	488	5.28	1.75	479	-.08	.490	-0.05	5.12	1.62	537	5.37	1.83	525	.25	.172	0.14
Mean intention	1.53	.88	488	1.51	.88	479	-.02	.671	0.02	1.61	.93	537	1.53	.92	525	-.08	.164	0.09
Mean plan	5.58	2.21	488	5.30	2.19	479	-.28	.050	-0.13	5.57	2.32	537	5.19	2.29	525	-.38	.003	-0.16
Mean attitude	1.45	.66	488	1.47	.66	479	.02	.573	-0.03	1.32	.70	537	1.35	.69	525	.03	.491	-0.04

^1^Includes confidence interval.

^2^These do not include imputed data as only measured at 6 months.

Notes. METS = metabolic equivalent of task, AUDIT = alcohol use disorder identification test FAEE = fatty acid ethyl esters, VAS = visual analogue scale technique, TTO = time trade-off technique SSC = single sample count, OR = odds ratio, BMI = body mass index, GP = general practitioner, A&E = accident and emergency

### Secondary outcomes

The intervention and control arms had statistically significant differences in levels of recreational drug use at 1-month and 6-month follow-up. Using estimations from the Single Sample Count Method, there were more recreational drug users in the intervention than the control arm at both time points (see Table 2). Similar (but not statistically significant) differences observed between arms in biochemical markers of recreational drug use at 1-month and 6-month follow-up.

At 6-month follow-up there was a statistically significant difference between the intervention and control arms in the number of non-elective hospital admissions (see Table 2), with fewer admissions in the intervention arm. There were no differences in other measures of health services usage.

At 1 and/or 6-month follow-up, participants in the intervention and control arms did not significantly differ on secondary measures of physical activity (i.e., sedentary activity, walking activity, university sports centre membership and usage), alcohol use (i.e., number of binge drinking days, AUDIT or biochemical markers of alcohol), smoking (i.e., number of cigarettes smoked by smokers, biochemical markers of smoking), health status, BMI (self-report and objective), and academic achievement (i.e., status and grades).

There were some significant differences between the arms on the social cognitive variables at 6-month follow-up. In particular, participants in the intervention arm reported stronger injunctive norms and perceptions of control, but weaker descriptive norms, than participants in the control arm for fruit and vegetable intake. Participants in the intervention arm were less likely than participants in the control arm to report that they had a clear plan for avoiding smoking and binge drinking.

#### Moderation and mediation analyses

Deprivation index, gender, nationality (UK vs. non-UK), and ethnicity (white vs. non white) did not moderate the effect of the intervention on the primary outcome variables. Mediation analyses were not conducted as the intervention and control arm only differed for smoking status and participants in the intervention arm were less likely to report having a clear plan to avoid smoking (contrary to hypothesis).

#### Engagement with the intervention

There was low engagement with the intervention. At 6-month follow-up only 383 of the 736 participants allocated to the intervention arm (52%) had completed the self-affirmation task, only 259 (35%) had accessed the health messages, only 8 participants (1%) had made a plan and only 15 participants (2%) downloaded the app.

#### Per protocol analysis

To assess the effect of engagement with the intervention three per protocol analyses were conducted that included all participants in the control arm (*n* = 558) and (i) only those participants in the intervention arm who had completed the self-affirmation profile (*n* = 383) and (ii) only those participants in the intervention arm who had completed the self-affirmation profile and accessed the health messages (*n* = 259). There were no changes in the effects of the intervention on primary outcome variables when these per protocol analyses were conducted.

### Comparison of dropouts versus completers

The 1,107 participants who completed at least one follow-up questionnaire differed from those who did not complete a follow-up questionnaire in nationality, *Ï‡*^2^ (1, *N* = 1445) = 19.91, *p* < .001, ethnicity, *Ï‡*^2^ (1, *N* = 1438) = 30.76, *p* < .001, gender, *Ï‡*^2^ (1, *N* = 1445) = 45.37, *p* < .001, baseline physical activity, *F*(1,1397) = 5.73, *p* = .017, BMI, *F*(1,1355) = 4.01, *p* = .045 and self-rated health status, *F*(1,1434) = 11.10, *p* = .001 (see Additional file 1: Table S1). Completers were more likely to be British, white and female, do less exercise at baseline, have lower BMI and report that they were in better health, than those who did not complete a follow-up questionnaire. The differences in dropout rates between the two arms of the trial approached statistical significance, *Ï‡*^2^ (1, *N* = 1445) = 3.40, *p* = .065 (25.4% intervention, 21.3% control).

## Discussion

The U@Uni RCT assessed the efficacy of a theory-based online health behaviour intervention (based on self-affirmation theory, the Theory of Planned Behaviour and implementation intentions) compared to a measurement only control. The intervention targeted fruit and vegetable intake, physical activity, alcohol consumption, and smoking in new students and was delivered during the transition to university. The RCT included two follow-up assessments, at 1 and 6-months after starting university. The intervention reduced rates of smoking at the 6-month follow-up (8.70% of participants in the intervention arm were smokers compared to 13.01% of the control arm) but did not affect fruit and vegetable intake, physical activity or alcohol consumption. These findings stand in contrast to the typical effects of online health behaviour interventions. For example, a meta-analysis by Webb et al. [3], found that Internet-based interventions tend to have small-sized effects on diet (*d* = 0.20), physical activity (*d* = 0.24), and alcohol consumption (*d* = 0.14), but a weaker effect on smoking behaviour (*d* = 0.07) than found in the current study (*d* = 0.25).

There are several possible reasons for the relatively weak effects found in the current trial. First, the U@Uni intervention targeted four health behaviours simultaneously. Webb et al. [3] found that interventions that targeted multiple health behaviours had smaller effects on health behaviour (*d* = 0.12) than those targeting a single health behaviour (*d* = 0.17). There are examples of successful multi-behaviour health interventions [4,5]; however, these focus solely on exercise and dietary behaviours that may be more complementary than those that also include health-risk behaviours such as alcohol consumption and smoking. Thus, the focus on multiple health behaviours may have diluted any intervention effects. This may have been amplified in the current intervention as participants had full control over the extent and type of information that they accessed on the intervention website. Second, there was low engagement with the intervention: only 383 (52%) of the 736 participants allocated to the intervention arm completed the self-affirmation task, 259 (35%) accessed the health messages, and 8 (1%) made a plan. Indeed, analyses of the social cognitive variables indicated that participants in the intervention arm were *less* likely than participants in the control arm to report that they had a clear plan for avoiding smoking and binge drinking. Low engagement may have been due to three issues. First, the baseline questionnaire was time consuming, as extensive measures were taken for the four health behaviours and additional variables (e.g., health status, recreational drug use, social cognitions, demographics, etc.). As a result, participants may have been fatigued by the time that they were directed to the intervention website. Second, there were technical glitches with the intervention software that made it easy for participants to disengage from the intervention. For example, after completing the baseline questionnaire, participants in the intervention arm were redirected to the intervention website where they were asked to complete some login information before completing the self-affirmation task. After completing this task, participants had to login again to the intervention website to view the health messages. There was evidence that participants dropped out at each of these stages. Third, owing to technical delays, invitation emails were sent to potential participants only two weeks before the start of the university semester (i.e., one week before “Freshers’ Week”). This likely coincided with a particularly busy time for many potential participants who may thus have failed to engage.

The secondary analyses revealed that, at least as estimated by the Single Sample Count Method [26], participants in the intervention arm were more likely to use recreational drugs than participants in the control arm at 1-month and 6-month follow-ups. Thus, there was some evidence that the intervention had undesired effects on non-targeted behaviours. Although, it is difficult to speculate how the intervention could lead to this undesired effect, one explanation for this effect is that participants in the intervention arm became more willing to take recreational drugs as they held a compensatory health belief that this unhealthy behaviour would be compensated for by their attempts to eat more fruit and vegetables, increase physical activity and reduce alcohol consumption [29]. No statistically significant differences were found between the two arms when biochemical markers of recreational drug use were considered, although the trend supported the results of the Single Count Method. However, given the low response rate to this aspect of the trial, this sample might be biased. The intervention had a mixed impact on the social cognitive variables. Participants in the intervention arm reported stronger injunctive norms and perceptions of control than participants in the control arm for fruit and vegetable intake. However, they also reported weaker descriptive norms for fruit and vegetable intake, and less clear plans for avoiding smoking and binge drinking. Finally, intervention participants reported fewer non-elective hospital admissions than the control arm at 6-month follow-up.

## Conclusions

The initial findings reported in this paper suggest that the U@Uni intervention may be effective at reducing smoking, but has little effect on other targeted behaviours (fruit and vegetable intake, physical activity and alcohol consumption). However, this conclusion is tempered by a number of important limitations, most notably, low engagement with the intervention that, in part, was due to a number of minor technical glitches with the software platform used to deliver the intervention. The mixed results, coupled with low engagement, suggest that further research is needed before the efficacy (or lack of efficacy) of the U@Uni intervention can be confirmed. In particular, future research should (i) reduce the length of the baseline questionnaire, (ii) send out invitation emails earlier, so that potential participants have more time to engage with the intervention before starting university, and (iii) ensure that participants’ experience of navigating through the intervention is optimised, so that they access more of the intervention material. A revised protocol for a repeat trial that incorporates >these features is available from http://www.sheffield.ac.uk/polopoly_fs/1.373196!/file/UatUni2_Protocol.pdf.

## Endnotes

^a^This follows the recommendations for clinical trials to only include participants who have completed one post-intervention measure [28]. If baseline scores are used as a covariate in the analysis, replacing missing data with baseline data is problematic. This is due to the inflated correlation between the dependent variable and the baseline data that reduces the variance. This is particularly problematic with a large dropout rate such as that in this study of 23% [27].

## Abbreviations

ANCOVA: Analysis of covariance; AUDIT: Alcohol use disorder identification test; BMI: Body mass index; GLF: General lifestyle survey; HSE: Health survey for England; IPAQ-SF: International physical activity questionnaire-short form; MANOVA: Multivariate analysis of variance; MET: Metabolic of equivalent task; QALY: Quality adjusted life years; RCT: Randomised controlled trial; TPB: Theory of Planned Behaviour.

## Competing interests

The authors declare that they have no competing interests.

## Author’s contributions

TE was responsible for the data analysis, wrote the first draft of this paper and finalized the paper after feedback from all other authors. PN, PS, PH, TLW, FC, AB, SJ, DN, AP, and PM designed and wrote the original proposal. PN, PS, PH, TLW and TE were primarily responsible for the design of the intervention content, FC and ASD for the design of the data structure/model and build of the website and mobile app, PM, AB and JK for the health economics evaluation, SJ for the statistical analysis plan, AP for the indirect prevalence estimation of drug use, AP and DN for the biochemical markers aspect of the study and IS for the hair sample analyses. All authors commented on drafts and approved the final version of the paper.

## Pre-publication history

The pre-publication history for this paper can be accessed here:

http://www.biomedcentral.com/1471-2458/14/563/prepub

## Supplementary Material

Additional file 1: Table S1 Baseline characteristics of the completers and dropouts table. Click here for file
