# Supplementary material for: A theory-based online health behaviour intervention for new university students (U@Uni): results from a randomised controlled trial
Source: BMC Public Health. 2014 Jun 5;14:563. doi: 10.1186/1471-2458-14-563 (PMC4067627; doi:10.1186/1471-2458-14-563)
Supplement: Additional file 1: Table S1 — Baseline characteristics of the completers and dropouts table. [file 1471-2458-14-563-S1.pdf]

Table S1 *Baseline Characteristics of the Completers and Drop Outs*

Variable	Completers				Drop Outs		
	% or mean	*SD*	*N*		% or mean	*SD*	*N*
**Condition**							
Intervention	74.59	-	549		25.41	-	187
Control	78.70	-	558		21.30	-	151
**Demographic**							
Nationality							
UK	79.47	-	863		20.53	-	223
Non UK	67.97	-	244		32.03	-	115
Ethnicity							
White British		-				-	
White other		-				-	
Mixed		-				-	
Asian and Asian British		-				-	
Black and black British		-				-	
Chinese		-				-	
Other		-				-	
Gender							
Female	82.94	-	700		17.06	-	144
Male	67.72	-	407		32.28	-	194
Age	18.88	2.60	1107		18.97	2.07	338
**Fruit and Vegetable Intake**							
Mean portions	6.45	5.09	1050		6.73	5.00	320
**Physical Activity**							
METS	3112.49	4121.35	1079		3801.25	5626.30	318
Mean hours sitting	341.40	178.70	291		341.84	174.14	959
**Alcohol Consumption**							
Mean total units in last 7 days	11.64	18.79	1107		11.11	18.11	337
Mean number of days binge drinking in last 7 days (drinkers only)	.98	1.07	693		1.15	1.15	179
Mean alcohol objective (FAEE)	2.10	2.31	106		.67	.59	2
**Smoking**							
Has smoked	75.46	-	406		24.54	-	132

Has never smoked	77.29	-	701		22.71	-	206
Current smokers	73.81	-	124		26.19	-	44
Not a current smoker	76.98	-	983		23.02	-	294
Mean cigarettes smoked per week	2.49	12.48	1105		3.77	16.65	337
Smoking objective (cotinine)	.47	.46	106		.03	.00	2
Smoking objective (nicotine)	8.34	18.86	106		.06	.08	2
**Other outcomes**							
EQ-5D-3L							
Mean health index scores from EQ-5D-3L(VAS)	.91	.14	1106		.91	.15	337
Mean health index scores from EQ-5D-3L (TTO)	.92	.13	1106		.92	.14	337
Mean EQ-5D-3L visual analogue scale	78.88	14.45	1100		75.82	15.65	336
BMI							
Mean BMI	22.04	3.69	1047		22.53	3.97	310
Mean BMI objective	22.18	3.88	106		21.43	.94	2
**Social cognition variables**							
Fruit & veg							
Self-efficacy	5.78	1.41	1100		5.67	1.47	336
Perceived control	5.61	1.54	1100		5.23	1.78	336
Intention	5.33	1.44	1100		5.29	1.53	336
Physical activity							
Self-efficacy	6.02	1.32	1104		5.97	1.38	337
Perceived control	5.67	1.42	1104		5.45	1.60	337
Intention	5.85	1.36	1104		5.90	1.40	337
Binge drinking							
Self-efficacy	5.51	2.15	1102		5.16	2.24	335
Perceived control	6.07	1.43	1102		5.77	1.64	335
Intention	3.14	1.96	1102		3.24	2.05	335
Smoking							
Self-efficacy	4.99	2.48	1103		4.68	2.53	336
Perceived control	6.51	1.29	1103		6.25	1.57	336

Intention	1.49	1.29	1103		1.72	1.54	336

Notes. METS = metabolic equivalent of task, FAEE = fatty acid ethyl esters
